# Physiological Reports 2021 Reviewers

**DOI:** 10.14814/phy2.15228

**Published:** 2022-03-28

The following people completed reviews for Physiological Reports in 2021. We thank these reviewers for their valuable service to the journal.

**Table:** 

Abbasi, Adeel
Abramochkin, Denis
Adams, David
Adams, Sean
Agergaard, Jakob
Ahmed, Mona
Akerman, Ashley
Alexander, John
Alexander, Matthew
Allu, Prasanna
Alonso, Laura
Alrefai, Waddah
Altamirano, Francisco
Alvarez de la Rosa, Diego
Amano, Toshiyasu
Amaral, Lorena
Amaral, Sandra Lia
Ambalavanan, Namasivayam
Andjelkovic, Anuska
Andrae, Johanna
Andrews, Chris
Ansdell, Paul
Archer, Stephen
Atabaki, Rabi
Attwell, David
Avoli, Massimo
Azab, Mohammad
Badenhorst, Claire
Bae, Jun Hyun
Baechler, Brittany
Bailey, E. Fiona
Bain, William
Baltzopoulis, Bill
Banovic, Marko
Bansal, Amar
Barrett, Kim
Basualto, Carla
Batista, Miguel
Batterham, Alan
Bauer, Natalie
Baynard, Tracy
Beckel, Jonathan
Behnke, Bradley
Behrens, Martin
Bell, Christopher
Bellicha, Alice
Beltrami, Fernando
Berg, Ronan
Bertolini, Anna
Beyer, Andreas
Bharucha, Adil
Bia, D.
Bianco, Antonino
Bock, Jonathan M.
Boedtkjer, Ebbe
Bogomolovas, Julijus
Bohosova, Julia
Bologna, Matteo
Booz, George
Borges, Cibele S.
Bosc, Laura
Boullosa, Daniel
Bowen, Robert
Boyd‐Shiwarski, Cary
Boye, Alex
Bramham, Kate
Brassard, Patrice
Breen, Ellen
Brewster, Luke
Brooks, Frank
Broomé, Michael
Brown, Alex
Brown, Angus
Brown, Mary Brth
Browning, Kirsteen
Brownstein, Callum
Brumberg, Joshua
Bunsawat, Kanokwan
Burns, Grace
Butler, Jane
Bye, Anja
Caccamo, Daniela
Caldwell, Jacob
Caldwell, Jessica
Calloe, Kirstine
Camic, Clayton
Cananzi, Sergio
Carattino, Marcelo
Cardozo, Licy Yanes
Cartee, Gregory
Case, Adam
Casimiro, Isabel
Castan‐Laurell, Isabelle
Ceyte, Hadrien
Challa, Siva Reddy
Chapman, Neil
Chaseling, Georgia
Chaudhary, Ketul
Cheang, Wai San
Chen, Bernadette
Chen, Wanjin
Christ, Torsten
Chun, Rene F.
Chung, Man‐Kyo
Ciccone, Anthony
Clarke, Lane
Clifford, Bethan
Coca, Steven
Collins, Helen
Collister, John
Cooke, Matthew
Coratella, Giuseppe
Cortassa, Sonia
Cui, Jian
Cummings, Kevin
Cuskelly, Annalisa
Czosnyka, Marek
D'Aout, Kris
Dalghi, Marianela
Dampney, RAL
Darby, Jack
David, Valentin
De Miguel, Carmen
de Waal, Koert
Debevec, Tadej
Dedkov, Eduard
Delpire, Eric
DeRuisseau, Lara
Di Virgilio, Francesco
Dibb, Katharine
Dinning, Phil
Dixon, Ian
Dobri, Georgiana
Dominici, Fernando
Dong, Zheng
Dongaonkar, Ranjeet
Donker, D. W.
Dossena, Silvia
Duhamel, Todd
Duncker, Dirk
Dyck, David
Eaton, Douglas
Ecelbarger, Carolyn
Economides, John
Edwards, Aurélie
Egan, Thomas
Elia, Maurizio
Elijovich, Fernando
Elmarakby, Ahmed Elmarakby
Elstad, Maja
Emery, Michael
Endre, Zoltan
Enoka, Roger
Erskine, Robert
Evans, David
Fabris, Luca
Falcone, John
Faraci, Frank
Federico, Aletti
Feng, Jun
Ferguson, Scott
Ferreri, Nicholas
Fessler, Michael
Figliuzzi, Marina
Fissell, William
Flanagan, Shawn
Forbes, Patrick
Fornazieri, Marco Aurelio
Fox, Henrik
Fraidenburg, Dustin
Fraidenraich, Diego
Franco, Martha
Fraser, Matthew
Friel, Kathleen
Frisk, Michael
Fröhlich, Michael
Fry, Andrew
Fujii, Naoto
Funahashi, Makoto
Garantziotis, Stavros
Garrud, Tess
Gartman, Eric
Gatterer, Hannes
Genders, Amanda
George, Sharon
Gibson, John
Gill, Andrew W
Gillum, Trevor
Gironacci, mariela
Glenny, Robb
Glickman, Ellen
Goggins, Bridie
Goldfarb, David
Gommer, Erik D.
Gong, Rujun
González‐Alonso, José
Gonzalez‐Vicente, Agustin
Gonzalez, Esther G.
Gonzalez, Javier
Gorr, Matthew
Gowdy, Kymberly M.
Grant, Stuart
Gray, Richard
Greaney, Jody
Groves, Alan
Guan, Zhengrong
Guidot, David
Gulbransen, Brian
Gurovich, Alvaro
Gutterman, David
Guyenet, PG
Gwin, Jess
Hackett, Peter
Hafed, Ziad M.
Halland, Magnus
Hamilton, Kirk L.
Hammers, David W
Hanakawa, Takashi
Hancox, Jules
Hantel, Constanze
Hanudel, Mark
Harding, Pamela
Hardy, Matthew
Harmancey, Romain
Hashemi‐Firouzi, Nasrin
Hassan, Mohammed H.
Hayashi, Koichiro
Heijman, Jordi
Heinrich, Erica
Hellsten, Ylva
Helms, My
Hester, Garrett
Hill, Cameron
Hill, Warren
Hillebrandt, David
Hippensteel, Joseph
Hjortdal, Vibeke
Hoedt, Emily
Hoeker, Gregory
Hoffman, Jay
Holz, George G.
Hong, Hui
Hook, Michelle
Hooman, Poor
Hoorn, Ewout
Hord, Jeffrey
Horiuchi, Masahiro
Hsia, Connie
Huang, Alex
Huang, Christopher
Huizinga, Jan
Hull, James
Hund, Thomas
Iemitsu, Motoyuki
Ilatovskaya, Daria
Ilg, Uwe
Imai, Yuuki
Imig, John
Ingram, Jennifer
Irani, Mudar
Ishikura, Keisuke
Ives, Stephen
Jaafar, Rami
Jamieson, Amanda
Jankowich, Matthew
Jankowski, Michael
Jarnicki, Andrew
Jeffrey, Miner
Jensen, Boye
Jeong, Jin Hee
Jepps, Thomas A
Jhamb, Manisha
Johansen, Peter
Johnson, Blair
Johnston, Richard
Jones, Samantha
Jurczak, Michael
Kaiko, Gerard
Kaiya, Hiroyki
Kaminsky, David
Kanaley, Jill
Katakam, Prasad
Katakami, Naoto
Kaufman, Marc
Kavazis, Andreas
Kawanishi, Noriaki
Kazi, Mirajul
Keely, Simon
Kelleher, Shannon L.
Keramidas, Michail
Kermorgant, Stephanie
Kirwan, Jennifer
Kitaoka, Yu
Kittisakmontri, Kulnipa
Klein, Janet
Kliment, Corrine
Kline, David
Klinger, James
Knight, John
Koga, Shunsaku
Kolta, Arlette
Kolwicz, Stephen C.
Koutakis, Panagiotis
Kratky, Dagmar
Krogh‐Madsen, Trine
Krüger, Karsten
Kumar, Rahul
Kumar, Rajiv
Laitano, Orlando
Lappe, Markus
Lau, Edmund
Layden, Brian
Lee, Yong‐Soo
Lefferts, Wesley
Levinthal, David
Li, Chunling
Liin, Sara
Lim, Hoong Sern
Lim, Yenkai
Lin, Fangming
Lin, Guiting
Lindinger, Michael
Linz, Benedikt
Lithgow, Hannah
Liu, Lai‐Ting
Liu, Ping‐Yen
Liu, Ruisheng
Liu, Su‐Mei
Livingston, Man
Lochmuller, Hanns
Loewen, Matthew
Low, David
Lu, Yong
Lucchesi, Pamela
Lund, Morten
Ma, Rong
MacLeod, Ken
Maguire, Greg
Maiaru, Maria
Malbert, Charles‐Henri
Malin, Steven
Mani, Alireza
Margolis, Lee
Marsh, Susan
Marshall, Andrew
Marshall, Janice
Martin, Brian
Martinot, Jean‐Benoit
Marzuca‐Nassr, Gabriel Nasri
Mashtoub, Suzanne
Massett, Michael
Matthews, Hugh
Matute‐bello, Gustavo
Maughnan, Ron
McArdle, Anne
McCafferty, Dominic
McCole, Declan F.
McCormick, James
McCulloch, Christopher A.
McDonnell, Adam
McGarr, Gregory
Mcguire, Scott
McKeever, Kenneth
Meade, Robert
Medelowitz, David
Meisner, Joshua
Melzer, Itshak
Mendham, Amy
Merry, Troy
Michael, Adrian C.
Milsom, Bill
Miyamoto, Toshiaki
Miyashita, Yosuke
Miyazaki, Teruo
Miyazato, Minoru
Mizuno, Masaki
Mobley, C. Brooks
Mohamed Zaki, Sherif
Monaghan, Sean
Moneim, Ahmed E. Abdel
Moore, Lorna
Moralez, Gilbert
Moretti, Antimo
Morgan, Paul
Morotti, Stefano
Mosqueira, Matias
Moss, Heather
Murach, Kevin
Murtha, Lucy
Musumeci, Giuseppe
Mutchler, Stephanie
Naeije, Robert
Naggar, Isaac
Naik, Jay
Nakata, Jun
Nakazato, Koichi
Naudin, Crystal
Nielsen, Eva
Nikolaev, Viacheslav
Nimmo, Alan
Nissen, Sarah Dalgas
Nistri, Andreas
Nono Nankam, Pamela
Nord, Andreas
Novak, Damen
Nygaard, Havard
O'Connor, Paul
O'Driscoll, Jamie
Ogoh, Shigehiko
Ogola, Benard
Olfert, Mark
Opdenakker, Ghislain
Osborn, Jeffrey
Overgaard, Kristian
Pal, Lubna
Pal, Sarit
Pallubinsky, Hannah
Palmer, Lawrence
Pan, Xiaoyue
Pao, Alan
Paoli, Antonio
Paquette, Max R.
Park, Song‐Young
Partiseti, Michel
Parvatiyar, Michelle
Patel, Sanket
Paterson, David
Patterson, Tiffany
Pawelczyk, James
Pearce, David
Pence, Brandt
Petersen, Kristian
Petrassi, Frank
Pironti, Gianluigi
Pla, Robin
Pockney, Peter
Polichnowski, Aaron
Pollock, David
Pollock, Ross
Potter, Michael
Power, Geoffrey
Praetorius, Helle
Prakoso, Darnel
Price, S. Russ
Prisby, Rhonda
Pun, Matiram
Raber, Jacob
Ranadive, Sushant
Randell, Scott
Rao, Mrinalini
Rasmussen, Peter
Ravn, Hanne Berg
Ray, Evan
Reese, Jeffrey
Reichner, Jonathan
Rein, Joshua
Reisi, Parham
Remme, Carol Ann
Ren, Jun
Ribet, David
Rissanen, Antti‐Pekka
Ritter, Megan
Rittner, Heike L.
Rizzo, Giuseppe
Roberts, Michael
Rodan, Aylin
Rodriguez‐Ortega, Elisa
Rogers, Natasha
Romero, Damian
Romero, Michael
Rosenberg, A.
Rosenberg, Alexander
Rougier, Jean‐Sébastien
Roumelioti, Maria‐Eleni
Roxburgh, Brendon
Rule, Andrew
Rullman, Eric
Ryan, Terence
Ryzhova, Larisa
Sadoshima, Junichi
Sakhaee, Khashayar
Salisbury, Amy
Salmon, Morgan
Sampath, Harini
Sankari, Abdulghani
Santamaria, Fidel
Sarathy, Jayashree
Schlegel, Amnon
Schoene, Robert
Schroeder, Elizabeth
Sciarretta, Sebastiano
Scott, Iain
Scully, Christopher
Secomb, Timothy
Segal, Mark
Selen, Luc P. J.
Senanayake, Tharindu
Sepehrimanesh, Masood
Sershen, Henry
Sevick, Eva
Shah, Ankit
Shanely, R. Andrew
Shantsila, Alena
Sharman, James E.
Sheikhbahaei, Shahriar
Shepard, Blythe
Shields, Michael
Shiwarski, Daniel
Shourijeh, Mohommed
Siebenmann, Christoph
Siegmund, Gunter
Sietsema, Kathy
Sikora, Justyna
Silva, Júlio
Simoneau, Martin
Simpson, Lydia
Sims‐Lucas, Sunder
Singh, Navrag
Singh, Reetu
Singla, Dinender
Sjoholm, Ake
Skelly, Deanne
Skytioti, Maria
Sluka, Kathleen A.
Smirl, Jonathan
Smiseth, Otto Armin
Smith, Frank
Smith, Jeffrey
Soares‐Jr, José Maria
Solverson, Patrick
Somers, Douglas
Somogyi, Peter
Sorrentino, Andrea
Spencer, Nick
Speth, Robert
Stacy, Mitchel R.
Stampas, Argyrios
Staunton, Caroline
Stembridge, Mike
Stengl, Milan
Stone, Brandon
Stults‐Kolehmainen, Matthew
Subramanya, Arohan
Sullivan, Jennifer
Sunny, Sini
Suzuki, Junichi
Sweazea, Karen
Swenson, Erik
Tadros, Melissa
Tai, Changfeng
Takegaki, Junya
Takeya, Kosuke
Talbot, Nick
Tamagnini, Francesco
Tan, Roderick
Tan, Xiao‐Di
Tanpowpong, Pornthep
Tardif, Nicolas
Tarnopolsky, Mark
Tawfeek, Hesham
Taylor, Andrew
Taylor, Jenna
Taylor, Victoria
Terentyev, Dmitry
Thamrin, Cindy
Theilig, Franziska
Thompson, John
Thomsen, Morten B.
Thornell, Ian
Tidball, James
Toledo, Frederico
Toney, Glenn
Trepiccione, Francesco
Trofimov, Alexander
Tsagareli, Merab G.
Tune, Johnathan
Vaira, Luigi
Van Helden, Dirk
Veeraraghavan, Rengasayee
Venetucci, Luigi
Ventetuolo, Corey
Veurveurt, Ingrid
Vianna, Lauro
Vieira‐Potter, Victoria
Vileigas, Danielle
Vogel, John
Voss, Andrew
Vyavahare, Naren
Wakabayashi, Hitoshi
Walker, Simon
Wang, Tiayanyi
Wang, Wen‐Hui
Warrington, Junie
Wasserstrom, J.
Webb, Clinton
Weinstein, Alan
Weissgerber, Tracey L.
Wimmers, Klaus
Wingo, Charles
Winn, Nathan
Witman, Melissa
Wong, Brett
Wright, David
Yang, Tianxin
Yaniv, Yael
Yarrow, Joshua
Ye, Xin
Yee, Jerry
Yin, Xiao‐Ming
Yoshimura, Naoki
Yoshino, Kohzoh
Yu, Aixi
Yuan, Ke
Zabbarova, Irina
Zampieri, Sandra
Zaniboni, Massimiliano
Zhang, Yanmin
Zhang, Delong
Zhang, Henggui
Zhang, Jibin
Zhao, Sumei
Zhou, Zhengfeng
Zignoli, Andrea
Ziouzenkova, Ouliana
Zubcevic, Jasenka

